# Integrated Transcriptomic Analysis Revealed Hub Genes and Pathways Involved in Sorafenib Resistance in Hepatocellular Carcinoma

**DOI:** 10.3389/pore.2021.1609985

**Published:** 2021-10-19

**Authors:** Xili Jiang, Wei Zhang, Lifeng Li, Shucai Xie

**Affiliations:** ^1^ Department of Radiology, The Second People’s Hospital of Hunan Province/Brain Hospital of Hunan Province, Changsha, China; ^2^ Department of Radiology, Changsha Central Hospital, Changsha, China; ^3^ Department of Critical Care Medicine, National Clinical Research Center for Geriatric Disorders, Xiangya Hospital, Central South University, Changsha, China

**Keywords:** hepatocellular carcinoma, hub gene, sorafenib resistance, integrated bioinformatics analysis, transcriptomic analysis

## Abstract

Hepatocellular carcinoma (HCC), a high mortality malignancy, has become a worldwide public health concern. Acquired resistance to the multikinase inhibitor sorafenib challenges its clinical efficacy and the survival benefits it provides to patients with advanced HCC. This study aimed to identify critical genes and pathways associated with sorafenib resistance in HCC using integrated bioinformatics analysis. Differentially expressed genes (DEGs) were identified using four HCC gene expression profiles (including 34 sorafenib-resistant and 29 sorafenib-sensitive samples) based on the robust rank aggregation method and R software. Gene ontology (GO) functional annotation and Kyoto Encyclopedia of Genes and Genomes (KEGG) pathway analysis were performed using the Database for Annotation, Visualization and Integrated Discovery (DAVID) online tool. A protein–protein interaction (PPI) network was constructed using the Search Tool for the Retrieval of Interacting Genes (STRING), and small molecules reversing sorafenib resistance were searched for using the connectivity map (CMAP) database. Pearson correlation and survival analyses of hub genes were performed using cBioPortal and Gene Expression Profiling and Interactive Analysis (GEPIA). Finally, the expression levels of hub genes in sorafenib-resistant HCC cells were verified using quantitative polymerase chain reaction (q-PCR). A total of 165 integrated DEGs (66 upregulated and 99 downregulated in sorafenib resistant samples compared sorafenib sensitive ones) primarily enriched in negative regulation of endopeptidase activity, extracellular exosome, and protease binding were identified. Some pathways were commonly shared between the integrated DEGs. Seven promising therapeutic agents and 13 hub genes were identified. These findings provide a strategy and theoretical basis for overcoming sorafenib resistance in HCC patients.

## Introduction

Hepatocellular carcinoma (HCC) is the most prevalent primary liver cancer, ranking sixth among the most common malignant tumors worldwide [1]. With approximately 8,41,000 new cases and 7,80,000 deaths in 2018, the incidence and mortality rates of HCC are increasing [2]. Variations in the incidence rate for HCC globally are attributed to differences in risk factors. In general, the incidence for HCC in developing countries is higher than that in developed countries. The majority of HCC patients (80%) are from East Asia and sub-Saharan Africa, where the major risk factors are the prevalence of chronic Hepatitis B virus and aflatoxin B1 [3], whereas, presence of Hepatitis C virus and alcohol overuse are the primary pathogenic factors for HCC in Europe, America and Japan [4].

Over the past decades, noticeable progress has been achieved towards the prevention, diagnosis, and treatment of HCC. Surgery, locoregional treatment, and systemic therapies have proven effective against HCC. Appropriate treatments for HCC depend on multiple factors, such as the tumor stage of HCC, performance status of patients, etc. [5]. Curative treatments including radiofrequency ablation, liver resection, and liver transplantation are more suitable for treating early or very early-stage cancer [5]. However, most patients with HCC present at an intermediate or advanced stage at the time of diagnosis. Eliminating the possibility of curative treatment, systemic treatment or palliative treatment is essential [4]. An in-depth evaluation of the pathogenesis of HCC has inferred that hepatocellular carcinogenesis and its progression are complex multi-step processes involving persistent genetic mutations. Therefore, research into and application of molecular targeted drugs for dysregulated genes associated with HCC are of great importance.

Since 2007, sorafenib, an effective first-line systemic therapy, has been approved for clinical application in patients with advanced-stage HCC and provides consistent survival benefits [6]. It is an orally active multiple-target tyrosine kinase inhibitor (TKI) that suppresses tumor angiogenesis and progression through various molecular targets, such as RAF/MAPK/ERK pathway, vascular endothelial growth factor receptor tyrosine kinases, platelet-derived growth factor receptor, fibroblast growth factor receptor, myeloid cell leukemia-1, FMS-like tyrosine kinase-3, receptor tyrosine kinase, and shugoshin-like 1 [7–11]. Robust evidence and clinical experiments inferred that sorafenib provides a cornerstone treatment by substantially extending the median overall survival of advanced-stage HCC patients [12,13]. With the approval of several immune-checkpoint inhibitors such as lenvatinib, regorafenib, cabozantinib, ramucirumab [14], targeted therapy provides a new strategy for HCC treatment.

However, acquired drug resistance becomes a vexing problem within 6 months of drug application. Studying the sorafenib resistance mechanism may aid in overcoming drug resistance and improve the targeted drug efficacy. Apart from epigenetic modifications, transport processes, or other mechanisms, gene disorders and signaling pathways are some common phenomena causing sorafenib resistance. Studies to identify critical genes and pathways might aid in reversing sorafenib resistance.

With the rapid development in sequencing technology, huge volumes of gene expression profiling data related to cancer were generated and uploaded to the Gene Expression Omnibus (GEO) database. Meanwhile, various analytical methods were applied for accurate identification of critically differentially expressed genes and pathways involved in cancer. As described previously [15], the robust rank aggregation (RRA) algorithm was used to analyze multiple gene lists based on different data platforms [16]. The statistical model of RRA assumes that all the genes in each dataset are randomly arranged. As a gene ranks higher in all datasets, its *p*-value gets lowered, which provides it with a greater possibility of getting converted to a differentially expressed gene (DEG).

The present study included 63 samples (34 sorafenib-resistant and 29 sorafenib-sensitive samples) from four datasets. R software was used to identify the DEGs in each dataset and RRA method was applied to obtain integrated DEGs. Subsequently, gene ontology (GO) term enrichment analyses and Kyoto Encyclopedia of Genes and Genomes (KEGG) pathway analyses of DEGs were performed on Database for Annotation, Visualization and Integrated Discovery online tool 6.8 (https://david.ncifcrf.gov/). The connectivity map (CMAP) database was used to search for small-molecule candidates which might reverse sorafenib resistance. Thereafter, the hub genes were inferred from protein–protein interaction (PPI) network of integrated DEGs and survival analysis was performed by generating overall survival curves in the gene expression profiling and interactive analysis (GEPIA). Finally, the expression levels of the top hub genes were verified via quantitative polymerase chain reaction in sorafenib-resistant hepatocellular carcinoma cells.

## Materials and Methods

### Microarray Data

Gene expression profiles of GSE62813, GSE73571, GSE151412, and GSE140202, were downloaded from the National Center for Biotechnology Information (NCBI) Gene Expression Omnibus (GEO) database (https://www.ncbi.nlm.nih.gov/geo/). GSE62813 and GSE73571 dataset are based on the platform of GPL6244 [HuGene-1_0-st] Affymetrix Human Gene 1.0 ST Array [transcript (gene) version], while the platforms of GSE151412 and GSE140202 dataset are GPL15520 Illumina MiSeq (*Homo sapiens*) and GPL20795 HiSeq X Ten (*Homo sapiens*), respectively (Table 1). Gene probe IDs were converted into international standard gene name using A Perl language command, and the gene expression data was normalized by the normalization Between Arrays function in the limma R package (http://www.bioconductor.org/) [17].

**TABLE 1:** Details of the GEO dataset.

Accession number	Sample	Platform	Sorafenib sensitive	Sorafenib-acquired resistant	Reference
GSE62813	HepG2 cells	GPL6244	3	7	van Malenstein H et al. (2013), Dekervel et al. (2016) [104,105]
GSE73571	Hepatospheres generated from tumors	GPL6244	3	3	Tovar et al. (2017) [106]
GSE140202	HepG2 and Huh7 cells	GPL20795	6	6	Wu et al. (2020) [107]
GSE151412	Huh7 and Hep3B cells	GPL15520	17	18	Wangensteen KJ et al. (No published)

### Screening for DEGs

The DEGs of each dataset were identified using limma R package V3.5.2 in R software with the cut-off criterion that adjusted *p*- value < 0.05 and |log_2_FC| > 1. Four gene lists, arranged according to log_2_FC value, were merged using the RobustRankAggreg R package (https://cran.rstudio.com/bin/windows/contrib/3.5/RobustRankAggreg_1.1.zip).[16] The integrated DEGs (upregulated and downregulated genes in sorafenib resistant samples compared sorafenib sensitive ones) were selected based on the cut-off criterion that *p*- value < 0.05 and |log_2_FC| > 0.5.

### Functional and Pathway Enrichment Analysis

As described previously [15], the Database for Annotation, Visualization and Integrated Discovery (DAVID, http://david.ncifcrf.gov) (version 6.8), a public online platform, is available for analyzing large-scale lists of genes or proteins and provides their biological information or characteristics for users. GO and KEGG pathway enrichment analysis were performed using the DAVID online tool. *p* < 0.05 was considered statistically significant.

### PPI Network Analysis

The Search Tool for the Retrieval of Interacting Genes (STRING) (https://string-db.org/) is commonly used to display the direct and indirect relationship of multiple proteins through forming PPI network [18]. The PPI network of integrated DEGs was exported and re-displayed by Cytoscape software (3.7.1), and the significant module was selected through the plug-in Molecular Complex Detection (MCODE) [19] app in Cytoscape software with the criterion that degree cut-off ≥ 2, node score cut-off ≥ 0.2, K-core ≥ 2, and max depth *D* = 100.

### Connectivity Map Analysis

The Connectivity Map (CMAP, http://www.broad.mit.edu/cmap/), a pattern-matching tool, was created to make disease–gene–drug connections [20]. By using the tool, users not only explore the mechanism of drug action, but also discover potential drugs for diseases through comparing its signature to the database to detect similarities [21]. The lists of upregulated and downregulated DEGs obtained from this study were submitted to CMAP to compare with the reference dataset. According to the enrichment of DEGs in the reference gene expression profile, a correlation score (−100∼100) was obtained; a positive number indicates that gene expression trend of the DEGs is similar to the reference gene expression profile. However, a negative number indicates that gene expression trend of the DEGs may be opposite to the reference gene expression profile.

### Pearson Correlation and Survival Analyses of Hub Genes

The hub genes were determined with degree ≥12. Pearson correlation analyses of hub genes were performed using the data of mRNA expression *z*-scores relative to all samples (log RNA Seq V2 RSEM) from the cBioPortal database for Liver Hepatocellular Carcinoma (TCGA, PanCancer Atlas, the genomic profiles including mutations, structural variant, putative copy-number alterations from GISTIC, mRNA expression z-scores relative to diploid samples). In addition, overall survival analysis of the top 13 hub genes was also accomplished on cBioportal. The tumor/normal differential expression analysis and prognostic value of hub genes were analyzed on GEPIA (http://gepia.cancer-pku.cn/), which is an interactive web application for gene expression analysis based on TCGA and GTEx data [22].

### Detection of the Expression Levels of Hub Genes in Sorafenib-Resistant Hepatocellular Carcinoma Cells

Sorafenib-resistant HCC cell line Huh7-SOR was generated by treating cells with a series of increasing concentrations ranging from 1 to 10 µM of sorafenib, and the concentration of sorafenib increased by 0.25 µM per cycle. Resistance indexes to sorafenib in Huh7 and Huh7-SOR cells were detected using CCK-8 assay [23]. Huh7-SOR cells were continuously cultured in the presence of 1 μM of sorafenib. RNA isolation and quantitative polymerase chain reaction (qPCR) analysis were conducted as described previously [24], and GAPDH was used as an endogenous expression control for mRNA. The primers of top 13 hub genes were designed and synthesized by Shanghai Bioengineering Co., Ltd. (Table 2).

**TABLE 2:** Primers list.

Gene symbol	Forward primer	Reverse primer	Amplicon (bp)
*SERPINA1*	CCG​TGA​AGG​TGC​CTA​TGA​TGA​AGC	AAG​AAG​ATG​GCG​GTG​GCA​TTG​C	115
*IGFBP1*	AGC​ACG​GAG​ATA​ACT​GAG​GAG​GAG	GTT​GGT​GAC​ATG​GAG​AGC​CTT​CG	129
*KNG1*	AAC​CTG​GCA​GGA​CTG​TGA​GT	CGT​ACT​GCT​CCT​CTT​CCC​CA	85
*TIMP1*	CCT​GGC​TTC​TGG​CAT​CCT​GTT​G	CGC​TGG​TAT​AAG​GTG​GTC​TGG​TTG	162
*APOA1*	ACA​GCG​TGA​CCT​CCA​CCT​TCA​G	TCC​ATC​TCC​TCC​TGC​CAC​TTC​TTC	187
*SPP1*	AGC​GAG​GAG​TTG​AAT​GGT​GC	TAA​CTG​TCC​TTC​CCA​CGG​CT	92
*IGFBP3*	CAA​GTA​GAC​GCC​TGC​CGC​AAG	GCT​GCT​GGT​CAT​GTC​CTT​GGC	85
*FBN1*	CAG​GAG​GAT​ACC​GCT​GTG​AAT​GC	GCC​GCT​TCT​GTC​CAG​TTC​GTA​G	176
*VCAN*	GAT​ACA​GCG​GAG​ACC​AGT​GTG​AAC	GGA​AGG​CAG​AGG​CAC​CTG​AAT​G	112
*MATN3*	AGG​AAA​CCT​TCT​GTG​CGC​TG	CTC​ACA​GTG​GTG​CTT​GCC​TT	95
*STC2*	ACG​GCC​TGG​TCA​CAT​GCT​CTC	TCC​TCC​TCC​TCC​TCT​TCC​TCC​TTC	151
*SERPINC1*	GAG​TGG​CTG​GAT​GAA​TTG​GAG​GAG	ATC​TCG​GCC​TTC​TGC​AAC​AAT​ACC	165
*APOB*	TGC​TCA​GTG​GAG​GCA​ACA​CAT​TAC	GCG​GAT​AGT​AGG​AGG​CGG​AGT​C	180

## Results

### Identification of Differentially Expressed Genes

**FIGURE 1:**
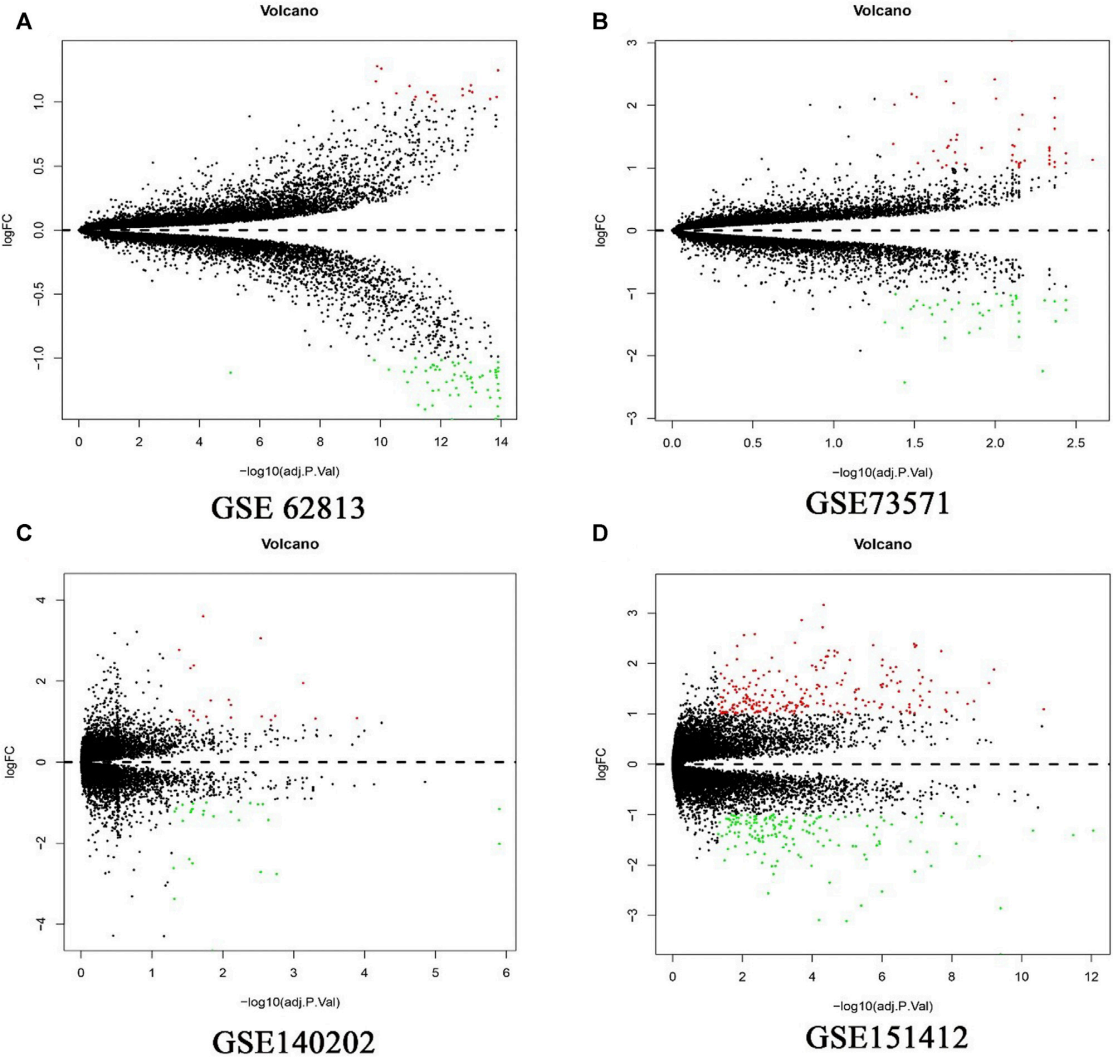
Differential expression genes between the two groups of samples in each dataset. **(A)** GSE62813, **(B)** GSE73571, **(C)** GSE140202, **(D)** GSE151412. The red dots represent the upregulated genes and the green dots represent the downregulated genes; the black spots represent genes with no significant difference in expression level.

In present study, 81 DEGs (20 upregulated and 61 downregulated), 91 DEGs (51 upregulated and 40 downregulated), 49 DEGs (27 upregulated and 22 downregulated), 402 DEGs (239 upregulated and 163 downregulated) were determined from four HCC expression profiles related to sorafenib resistance (Table 3 and Figure 1). After integrated analysis, a total of 165 DEGs (66 upregulated and 99 downregulated) were selected (Supplementary Table S1).

**TABLE 3:** Number of upregulated and downregulated DEGs in each dataset.

GEO	Sample	Number of DEGs	Number of upregulated	Number of downregulated
GSE62813	HepG2 cells	81	20	61
GSE73571	Hepatospheres generated from tumors	91	51	40
GSE140202	HepG2 and Huh7 cells	49	27	22
GSE151412	Huh7 and Hep3B cells	402	239	163

### Functional and Pathway Enrichment Analysis

**FIGURE 2:**
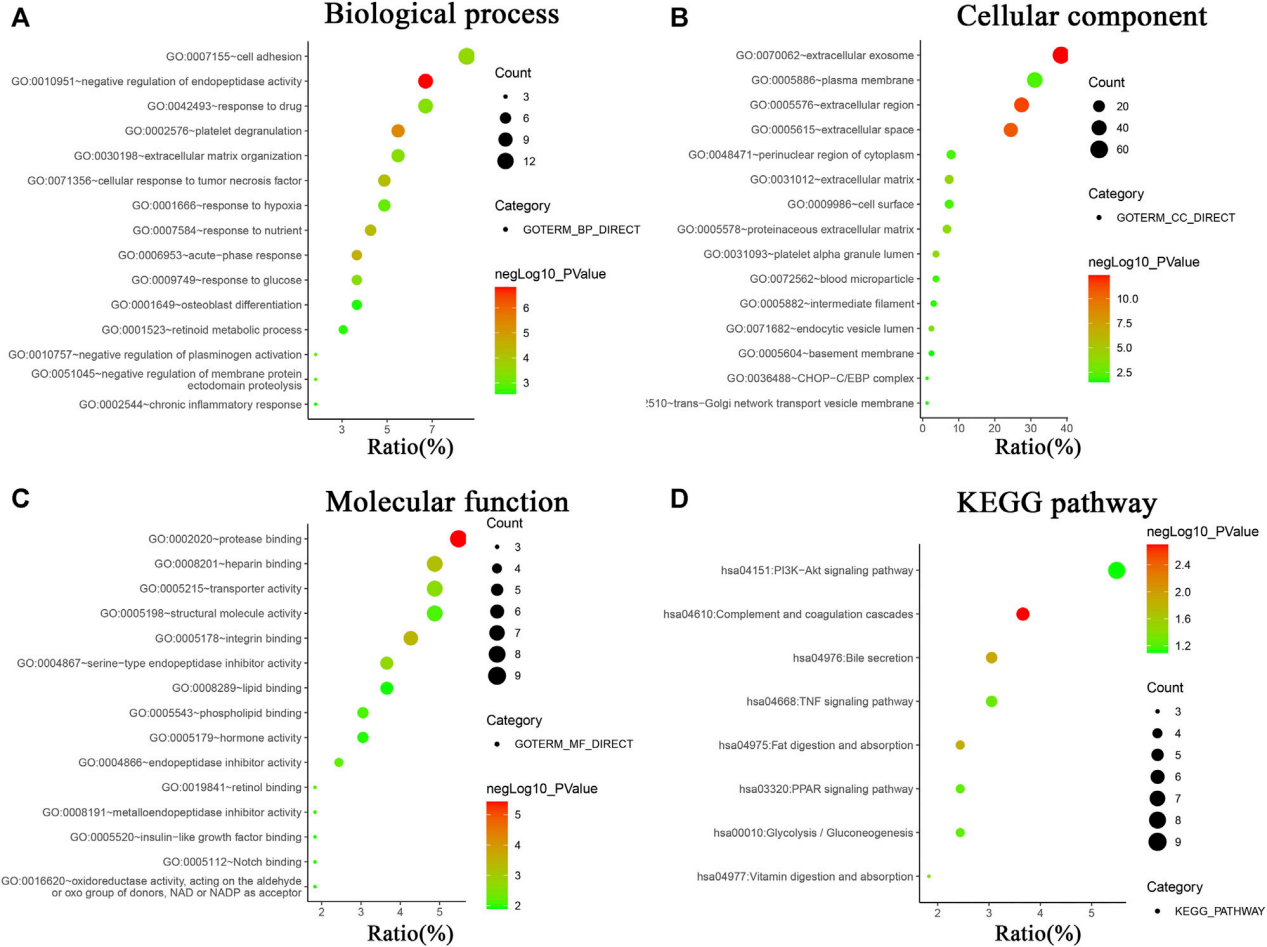
GO analysis and KEGG pathway enrichment analysis of DEGs using online database DAVID. **(A)** Biological processes of GO analysis, **(B)** cellular components of GO analysis, **(C)** molecular functions of GO analysis, **(D)** KEGG pathway enrichment analysis.

In order to systematically and comprehensively assess biological information of the integrated DEGs, GO enrichment analysis and KEGG pathway enrichment analysis were conducted using online database DAVID 6.8. GO enrichment analysis is made up of biological process group (BP), cellular component group (CC), and molecular function group (MF). As shown in Figure 2 and Supplementary Table S2, the 165 DEGs were mainly enriched in 95 biological process terms, 22 cell component terms, 32 molecular function terms, and 9 KEGG pathway terms. Moreover, the DEGs were participated in multiple biological process, especially focusing on negative regulation of endopeptidase activity (11 genes), platelet degranulation (9 genes), acute-phase response (6 genes), response to nutrient (7 genes), and cellular response to tumor necrosis factor (8 gene). For the CC, the DEGs were particularly enriched in extracellular exosome (63 genes), extracellular region (45 genes), extracellular space (40 genes), extracellular matrix (12 genes), and platelet alpha granule lumen (6 genes). For the MF, the DEGs were associated with protease binding (9 genes), integrin binding (7 genes), heparin binding (8 genes), serine-type endopeptidase inhibitor activity (6 genes), and transporter activity (8 genes).

KEGG pathway enrichment analysis revealed the DEGs were mainly involved in PI3K-Akt signaling pathway (9 genes), complement and coagulation cascades (6 genes), bile secretion (5 genes), TNF signaling pathway (5 genes), fat digestion and absorption (4 genes), PPAR signaling pathway (4 genes), Glycolysis/Gluconeogenesis (4 genes), and vitamin digestion and absorption (3 genes).

### Specific Drug Screening From CMAP Database to Relieve Sorafenib Resistance

In order to discover small molecules that have the potential to reverse the resistance of sorafenib, the 165 DEGs were submitted to CMAP for analysis with the cut-off criterion that the number of repeat experiment times≥ 4, the mean ≤−0.4 and *p*-value < 0.05. Seven small-molecule candidates were identified, including pentetrazol, hesperidin, digoxin, mebeverine, cinnarizine, sulfadiazine, sulfametoxydiazine (Table 4).

**TABLE 4:** Small molecule agents with potential abilities to overcome sorafenib resistance of HCC were identified by CMAP database.

Rank	Cmap name	Mean	*n*	Enrichment	*p*	Specificity	Percent non-null
2	pentetrazol	−0.461	4	−0.865	0.00062	0	75
3	hesperidin	−0.452	4	−0.822	0.00193	0	75
15	digoxin	−0.554	4	−0.726	0.01162	0.051	75
32	mebeverine	−0.438	4	−0.654	0.03348	0.0479	75
39	cinnarizine	−0.414	4	−0.635	0.04297	0.0514	75
40	sulfadiazine	−0.411	5	−0.568	0.04522	0.0737	60
42	sulfametoxydiazine	−0.442	4	−0.63	0.04597	0.1214	75

### PPI Network Analysis to Identify Hub Genes

**FIGURE 3:**
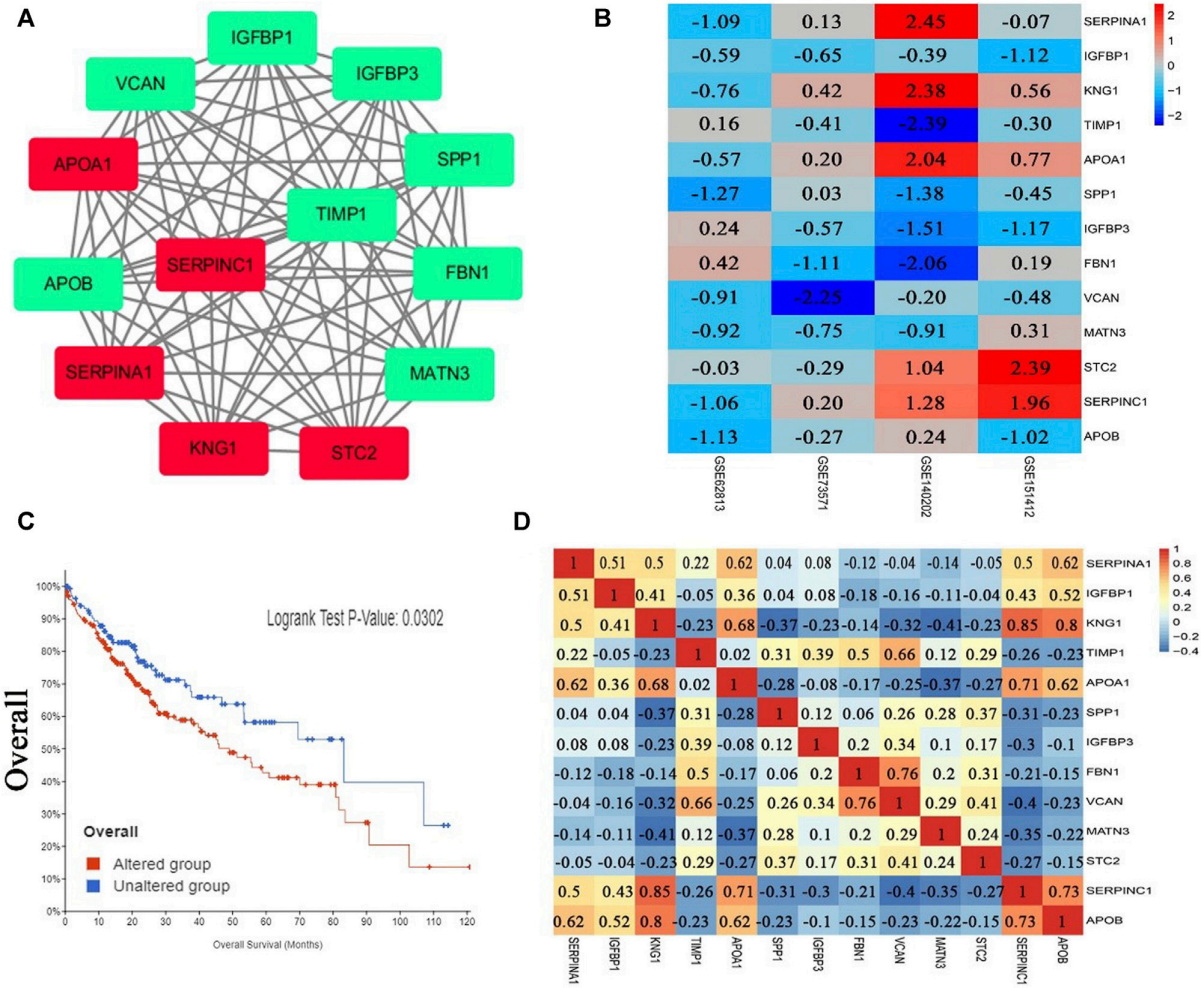
PPI network, Pearson correlation and overall survival analysis of top 13 hub genes. **(A)** The most significant module of hub genes from PPI network (the red represents log FC *>* 0, the green represents log FC *<* 0). **(B)** Heatmap of the expression levels of top 13 genes in 4 datasets. **(C)** Overall survival analysis of top 13 hub genes (altered group = 227, unaltered group = 144). **(D)** Pearson correlation analysis of top 13 hub genes.

After analysis, 36 genes were filtered out, and the remaining 129 genes (53 upregulated and 76 downregulated) formed the PPI network, which contained 129 nodes and 401 edges (Supplementary Figure S1, Supplementary Tables S3, S4). Using MCODE for automatic screening, 13 central node genes with 12° (i.e., each node has more than 12 connections/interactions) or more were recognized as hub genes. The top 13 hub genes were as follows: *SERPINA1, IGFBP1, KNG1, TIMP1, APOA1, SPP1, IGFBP3, FBN1, VCAN, MATN3, STC2, SERPINC1,* and *APOB*(Table 5 and Figure 3A). The expression levels of the top 13 hub genes from 4 datasets were showed in a heatmap. As shown in Figure 3B, *IGFBP1* and *VNAN* were downregulated in sorafenib resistant samples of 4 datasets, *TIMP1, SPP1, IGFBP3, MANT3,* and *APOB* were downregulated in sorafenib resistant samples of three datasets. However, *KNG1, APOA1,* and *SERPINC1* were upregulated in sorafenib resistant samples of three datasets.

**TABLE 5:** The top 13 most degree values hub genes between sorafenib sensitive and acquired sorafenib resistant HCC cells.

Gene symbol	Official full name	*p*-value	logFC	Degree	Up/Down
*SERPINA1*	serpin family A member 1	0.007168	0.611398	12	up
*IGFBP1*	insulin like growth factor binding protein 1	2.14E-05	−0.68358	12	down
*KNG1*	kininogen 1	0.007819	0.596	12	up
*TIMP1*	TIMP metallopeptidase inhibitor 1	0.009772	−0.73532	12	down
*APOA1*	apolipoprotein A1	0.010671	0.609391	12	up
*SPP1*	secreted phosphoprotein 1	0.000241	−0.76828	12	down
*IGFBP3*	insulin like growth factor binding protein 3	1.72E-05	−0.75524	12	down
*FBN1*	fibrillin 1	6.36E-05	−0.64256	12	down
*VCAN*	versican	0.000293	−0.9605	12	down
*MATN3*	matrilin 3	1.95E-05	−0.56723	12	down
*STC2*	stanniocalcin 2	0.001037	0.776586	12	up
*SERPINC1*	serpin family C member 1	0.000293	0.595693	12	up
*APOB*	apolipoprotein B	0.001625	−0.54464	12	down

### Pearson Correlation and Overall Survival Analysis of Hub Genes

The results of Pearson correlation analysis indicated that strong positive correlations were observed in the following hub genes (Figure 3D): *SERPINA1* with *APOA1, SERPINA1* with *APOB, KNG1* with *APOA1, KNG1* with *SERPINC1, KNG1* with *APOB, TIMP1* with *VCAN, APOA1* with *SERPINC1, APOA1* with *APOB, FBN1* with *VCAN*, and *SERPINC1* with *APOB*. Moderate positive correlations were observed in the following hub genes: *SERPINA1* with *IGFBP1, SERPINA1* with *KNG1, SERPINA1* with *SERPINC1, IGFBP1* with *KNG1, IGFBP1* with *SERPINC1*, *IGFBP1* with *APOB, TIMP1* with *FBN1*, and *STC2* with *VCAN*. However, moderate negative correlations were observed for *KNG1* with *MATN3*, and *FBN1* with *SERPINC1*. In addition, significant difference was observed between groups of cases with and without alteration(s) in top 13 hub genes (logrank test *p*-value, 0.0302) from the overall survival analysis generated from the cBioPortal database for Liver Hepatocellular Carcinoma (TCGA, PanCancer Atlas, the genomic profiles including mutations, structural variant, putative copy-number alterations from GISTIC, mRNA expression z-scores relative to diploid samples) (Figure 3C).

### The Expression Levels of Hub Genes in Liver Hepatocellular Carcinoma Patients and its Potential Prognostic Efficacy

**FIGURE 4:**
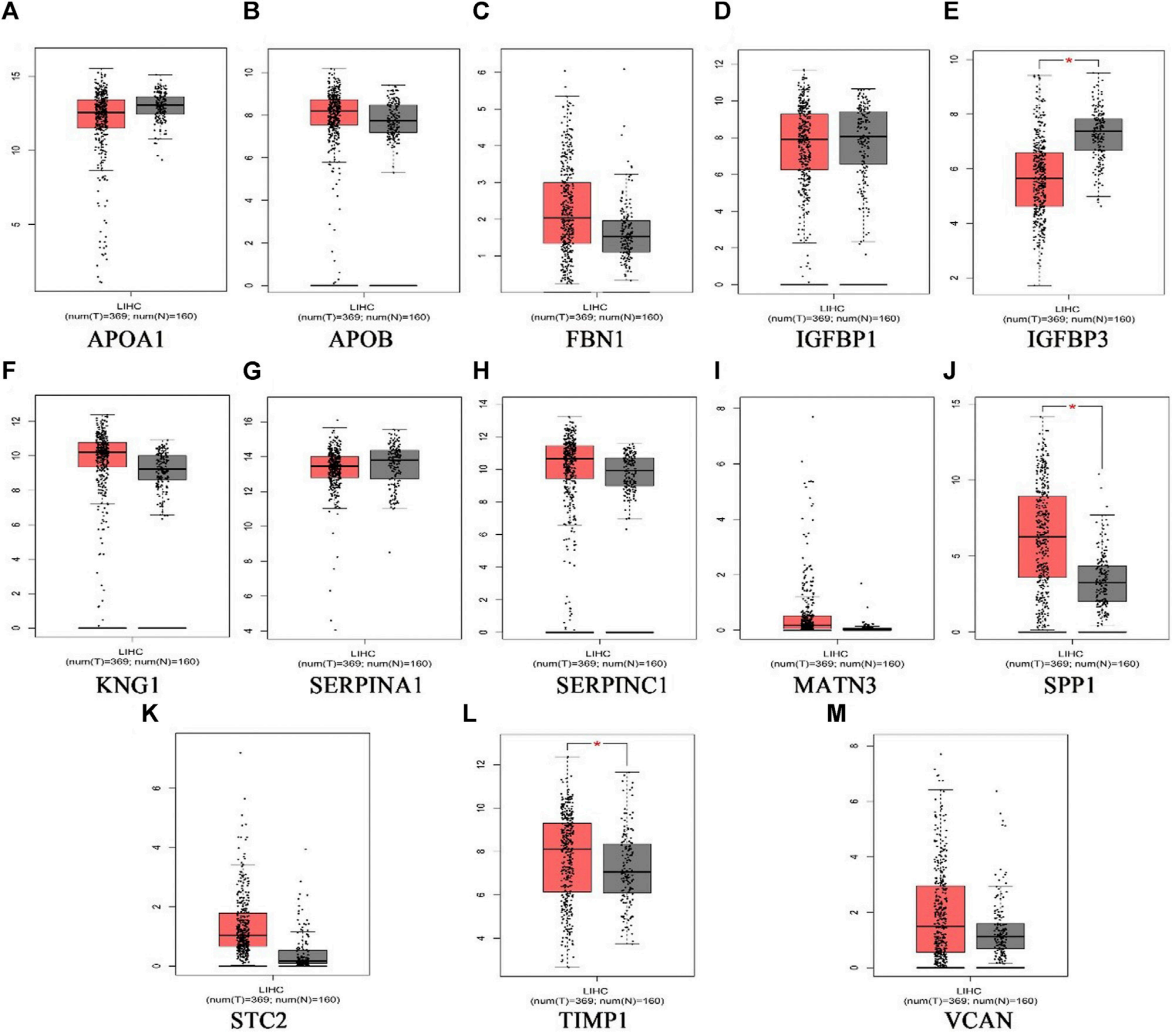
The expression level of hub genes in liver hepatocellular carcinoma patients. **(A)**APOA1, **(B)**APOB, **(C)**FBN1, **(D)**IGFBP1, **(E)**IGFBP3, **(F)**KNG1, **(G)** SERPINA1, **(H)** SERPINC1, **(I)** MATN3, **(J)** SPP1, **(K)** STC2, **(L)** TIMP1, **(M)** VCAN. *p* < 0.05 (*). (the red represents tumor group, the grey represents normal group).

**FIGURE 5:**
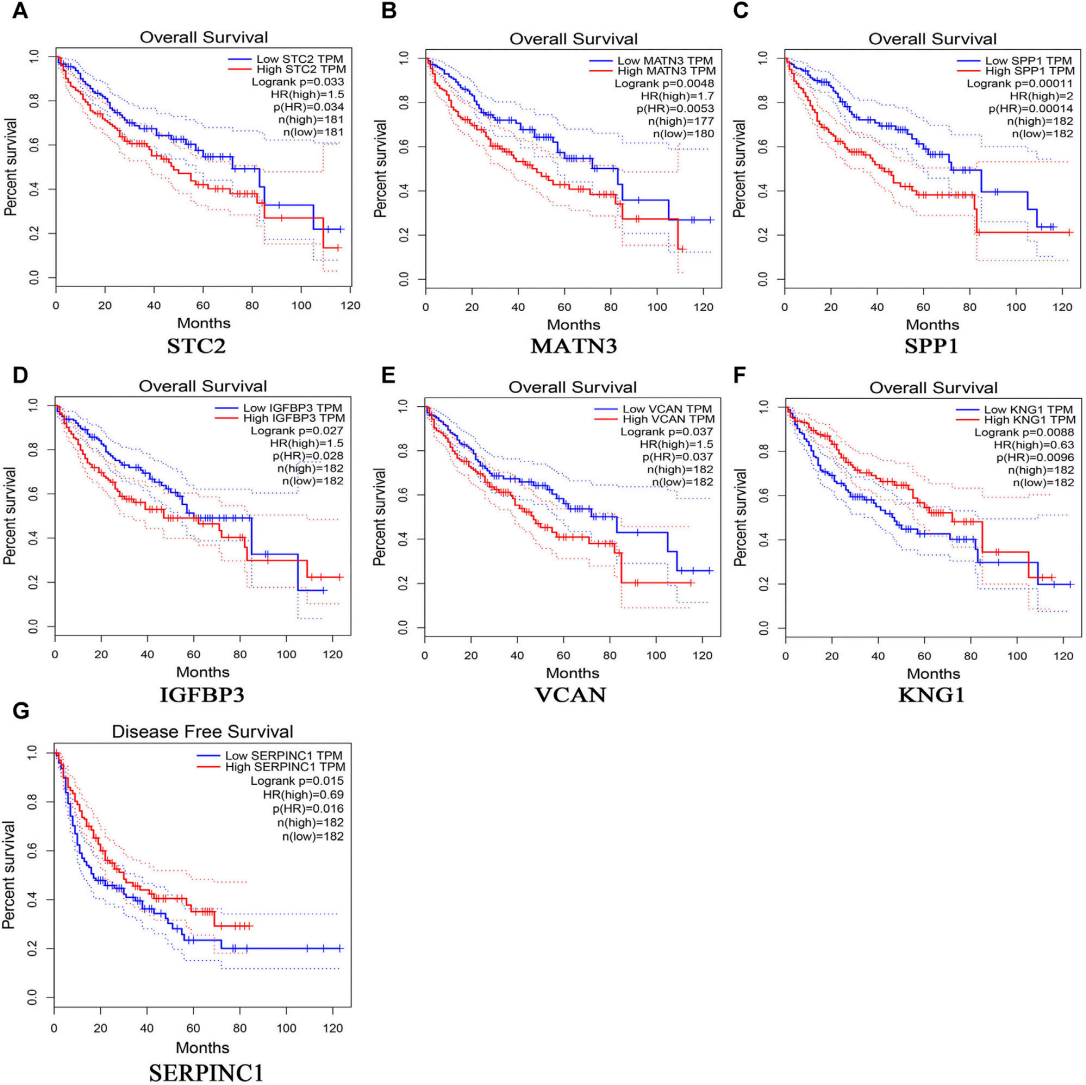
Overall survival analyses and disease free survival of single hub genes were generated from the GEPIA. Overall survival analyses: **(A)** STC2, **(B)** MATN3, **(C)** SPP1, **(D)** IGFBP3, **(E)** VCAN, **(F)** KNG1; Disease free survival, **(G)**SERPINC1.

369 liver cancer and 160 normal tissues from TCGA/GTEx datasets were included tumor/normal differential expression analysis using the GEPIA. Compared with normal tissues, the expression levels of *TIMP1* and *SPP1* in liver hepatocellular carcinoma tissues (LIHC) were significantly upregulated, while *IGFBP3* was significantly downregulated (Figure 4). Prognostic value of single hub gene was assessed through survival plots of the overall survival generated from the GEPIA. As shown in Figures 5A–F, HCC patients with *STC2, MATN3, SPP1, IGFBP3*, and *VCAN* up-regulation showed worse overall survival. Nonetheless, patients with *KNG1* down-regulation showed worse overall survival. HCC patients with *SERPINC1* down-regulation showed worse disease free survival (Figure 5G).

### The Expression Levels of Hub Genes in Sorafenib-Resistant Hepatocellular Carcinoma Cells

**FIGURE 6:**
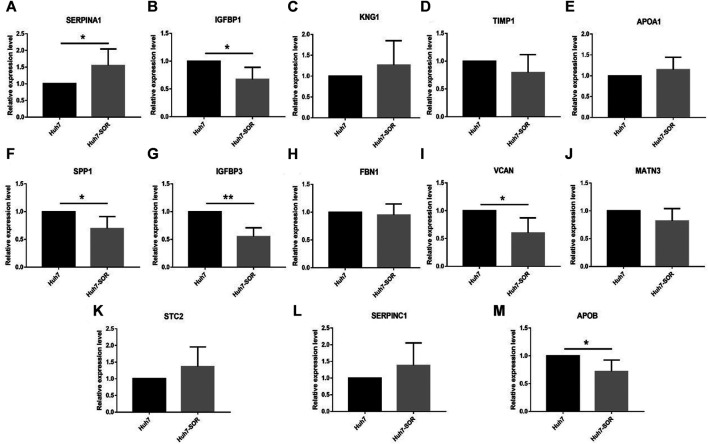
Detection of hub genes in sorafenib-resistant hepatocellular carcinoma cells by qRT-PCR. **(A)** SERPINA1, **(B)** IGFBP1, **(C)** KNG1, **(D)** TIMP1, **(E)** APOA1, **(F)** SPP1,**(G)** IGFBP3, **(H)** FBN1, **(I)** VCAN, **(J)** MATN3, **(K)** STC2, **(L)** SERPINC1, **(M)** APOB. *p* < 0.05 (*), *p* < 0.01 (**).

After cells were treated by sorafenib, the IC50 of sorafenib in Huh7-SOR cells was signifcantly higher than that of sorafenib in Huh7 cells (12.9 ± 1.4 μM vs. 7.1 ± 1.6 μM). The expression levels of 13 hub genes were measured by PCR in Huh7 and Huh7-SOR cells. Figure 6 and Supplementary Table S5 showed that six genes (*SERPINA1, IGFBP1, SPP1, IGFBP3, VCAN, and APOB*) were significantly increased or decreased between Huh7 and Huh7-SOR cells (*p* ≤ 0.05), whereas the remaining genes were moderately changed.

## Discussion

In this study, we identified 165 sorafenib resistance-related DEGs in 63 samples (29 sorafenib-sensitive samples and 34 sorafenib-resistant) from four datasets using RRA statistical model. GO and KEGG pathway enrichment analysis revealed that DEGs were intricately involved in several biological processes and pathways that play a vital role in drug resistance in HCC. For the CC, the DEGs of present study were particularly enriched in extracellular. Extracellular matrix, an important component of tumor microenvironment, is a complex network surrounding the cells [25]. Exosomes, released by multiple cells types, contain various types of protein, lipids, nucleic acids (DNA, mRNA, and miRNA) and other molecules [26]. Emerging evidence suggests ECM and exosomes have great potential to support the development of drug resistance in HCC cells [27,28]. In additon, inhibition of the PI3K/Akt signaling pathway, TNF-α, PPARγ, and PPARδ reverses sorafenib resistance in HCC [29–35]. Elevated glycolysis was observed in sorafenib-resistant HCC cells, and downregulating the key glycolytic enzymes 6-phosphofructo-2-kinase/fructose-2,6-biphosphatase (encoded by PFKFB3) or pyruvate kinase muscle isozyme M2 re-sensitizes HCC cells to sorafenib [31,36,37].

Using the CMAP database, seven small-molecule candidates were identified as the most promising therapeutic agents which have the potential to reverse sorafenib resistance. Evidence shows that pentetrazol, hesperidin, and digoxin have the ability to change the sensitivity of tumor cells towards chemotherapeutic drugs. Targeting exosome biogenesis and release have important clinical significance in cancer treatment. Pentetrazol was identified as an activator of exosome biogenesis and/or secretion in prostate cancer cells [38]. Hesperidin sensitized Ramos cells to doxorubicin-induced apoptosis [39], and co-chemotherapy of doxorubicin and hesperidin showed the ability to overcome resistance of doxorubicin by suppressing P-glycoprotein expression in Michigan Cancer Foundation-7 (MCF-7)-resistant doxorubicin cells [40]. Co-administration of hypoxia inducible factor inhibitor and cytotoxic chemotherapy overcame the resistance of breast cancer stem cells to paclitaxel or gemcitabine [41]. There is no evidence showing that mebeverine, cinnarizine, sulfadiazine, and sulfamethoxydiazine were involved in regulating the sensitivity of chemotherapy resistance. Nevertheless, further *in vivo* and *in vitro* experiments are still needed to validate their activity in HCC sorafenib resistance and to explore additional potential molecular mechanisms.

The most significant module was selected from the PPI network and 13 hub genes were identified. After analysis in the GEPIA, *TIMP1*, *SPP1*, *IGFBP3* were dysregulated in liver hepatocellular carcinoma patients. In addition, HCC patients with *STC2*, *MATN3, SPP1*, *IGFBP3*, *VCAN*, and *KNG1* dysregulation showed worse overall survival. Moreover, *SERPINA1, IGFBP1, SPP1, IGFBP3, VCAN, and APOB* were significantly increased or decreased between Huh7 and Huh7-SOR cells in sorafenib-resistant HCC cells. *SPP1* and *STC2* have been found to be involved in chemotherapy resistance in HCC [42,43]. In another bioinformatics analysis, *SERPINA1* was confirmed as the key gene from GSE109211 in sorafenib-resistant HCC cells [44].

The involvement of *IGFBP1*, *TIMP1*, *SPP1*, *IGFBP3,* and *STC2* in mediating chemotherapy resistance in various cancers have been extensively studied. Through a genome-wide RNA interference screen, *IGFBP1* was identified as one of the novel genes causing cellular resistance to neratinib [45]. Molecular mechanistic understanding has revealed that *IGFBP1* is a key component of G protein-coupled estrogen receptor 1 (encoded by *GPER1*) which regulated the sensitivity of breast cancer cells to tamoxifen [46,47], and the resistance induced by RG7388 (MDM2 inhibitor) in glioblastoma [48]. Another study showed that reduction in insulin-like growth factor I (encoded by *IGF-I*) mediated part of the starvation-dependent differential stress resistance [49].

Elevated tumor tissue *TIMP1* levels were significantly associated with a poor response to chemotherapy [50–52]. *TIMP1* deficiency increases either tumor cell sensitivity to chemotherapy or TNF-α-induced apoptosis [53–55]. A change in *TIMP1* expression could mediate resistance to gemcitabine in pancreatic cancer [56,57], to fulvestrant in MCF-7 human breast cancer cells [58], to platinum in epithelial ovarian cancer [59], to carboplatin in lung cancer [60], and to antiestrogen in breast cancer [61]. Furthermore, recombinant fusion protein linking TIMP1 to glycosylphosphatidylinositol anchor enhances tumor sensitivity to doxorubicin [62]. Moreover, another study using bioinformatics analysis revealed that *TIMP1* has an association with lapatinib resistance [63].

Osteopontin (*OPN*), also known as *SPP1*, was identified as a candidate drug resistance biomarker in ovarian [64], pancreatic [65], and metastatic castration-resistant prostate cancers [66]. Upregulation of *OPN* expression contributed to cisplatin (DDP) resistance in cervical cancer cells [67,68], cetuximab-resistance in head and neck squamous cell carcinoma [69], second-generation epidermal growth factor receptor (EGFR)-TKI resistance in lung cancer [70], leukemic stem cell chemoresistance in acute myeloid leukemia [71], chemoresistance of HCC via autophagy [42], and chemotherapy resistance of mouse WAP-SVT/t breast cancer cells [72]. Disruption of *OPN* sensitized chemotherapy in experimental mammary tumors and metastatic breast cancer [73], and *OPN* knockdown reduced resistance to some drugs as manifested via increase in cell death [74]. In clinical trials, *OPN* expression was associated with the efficiency of neoadjuvant chemotherapy (NACT) in breast cancer treatment [75].

*IGFBP3* is a specific biomarker associated with drug resistance of neuroblastoma cells to doxorubicin [76], gastric cancer cells to cisplatin [77], human epidermal growth factor receptor 2 (*HER2*) positive breast cancer to trastuzumab [78], lung adenocarcinoma harboring an EGFR mutation to afatinib [79], ovarian cancer to cisplatin [80], and non-small cell lung cancer (NSCLC) cells to docetaxel or gemcitabine [81]. Loss in *IGFBP3* expression increased the response of the U251 human glioblastoma cell line to CA 125 [82], and enhanced antitumor action of DZ-50 in prostate cancer [83]. However, the downregulated expression of *IGFBP-3* mediated the resistance to gefitinib in A431 squamous cancer cells [84], reduced the apoptosis of antiestrogen-resistant breast cancer cells to ICI 182,780 [85], increased the resistance to trastuzumab therapy in HER2 positive breast cancer [86], and reduced tumor sensitivity to molecular-targeted therapies in NSCLC [87]. In addition, growth factor sequestration by engineered *IGFBP-3* enhanced the activity of EGFR inhibitors [88].

Upregulated *STC2* was involved in drug resistance in HCC by increasing P-glycoprotein and B-cell lymphoma 2 protein expression levels [43], and it imparted resistance against EGFR tyrosine kinase inhibitors in lung cancer [89], resistance of cervical cancer cells to cisplatin [90], and induced oxaliplatin resistance in colorectal cancer cells [91]. Increased *STC2* expression induced by anti-vascular endothelial growth factor antibody therapy was observed in colon cancer, whereas the role of *STC2* is still unclear [92]. Patients who received first-line endocrine therapy with low-level expression of *STC2* showed poor outcome [93].

Several studies have showed that *SERPINA1*, *APOA1*, *VCAN*, and *APOB* mediated the development of cancer chemotherapy resistance. The differential expression of *SERPINA1* has been associated with platinum resistance in human epithelial ovarian cancer [94], CDDP resistance in gastric cancer (GC) [95], and tamoxifen resistance in breast cancer [96]. APOA1 was identified as a candidate drug resistance biomarker for ovarian cancer via 2D-gel proteomics [97]. *APOA1* was found to be upregulated in the drug respondent group when compared to the drug-resistant group of HIV-1 patients treated with first-line antiretroviral therapy [98]. *VCAN* was upregulated in spiky (CRC cell line) that was resistant to growth inhibition of cetuximab, and VCAN staining strongly correlated with reduced survival in colorectal cancer [99]. Versican V1 overexpression in lymphoid cell lines enhanced their sensitivity to doxorubicin and gemcitabine [100]. Serum levels of APOB could predict responses to NACT and relapse-free survival in advanced breast cancers patients [101]. However, no research has shown that *KNG1*, *FBN1*, *MATN3*, and *SERPINC1* play an important role in cancer chemotherapy resistance.

In accordance with our findings, the identification of genes and signaling pathways related to sorafenib resistance has also been found using bioinformatics methods in several studies[44,102,103]. Using the web tool GEO2R, GSE73571, and GSE109211 datasetwere analyzed. Sorafenib resistance-related DEGs (1,319 in total; 593 upregulated and 726 downregulated in sorafenib resistant samples compared sorafenib sensitive ones) were obtained, and the eight hub genes were identified from the GSE73571 dataset [103]. 164 sorafenib resistance-related DEGs (121 upregulated and 43 downregulated in sorafenib resistant samples compared sorafenib sensitive ones) were identified and nine hub genes were confirmed as key genes from the GSE109211 dataset [44]. In another study on the identification of microRNAs and transcription factors related to sorafenib resistance, GSE73571 was used and 827 significant DEGs were obtained using the “limma” package of R language [102].

Compared to the previous three studies, four GEO datasets comprising 61 samples were integrated and analyzed to obtain DEGs using the RRA method in present study. These four datasets included three HCC lines (Huh7, Hep3B, and HepG2) and hepatospheres generated from tumors (Huh7 cells) based on three different platforms (GPL6244, GPL20795, and GPL15520). Therefore, the hub genes identified in the present study are more reliable and comprehensive. Furthermore, we have also identified some small-molecule candidates that may overcome the resistance to sorafenib. However, the number of samples used in present study is relatively small, and the type of sample included is single (only HCC cell samples). The mechanisms and functions of DEGs in the resistance of sorafenib have not been further explained.

### Conclusion

In summary, by conducting an integrated bioinformatics analysis using multiple datasets, we identified some hub genes and pathways involved in sorafenib resistance for HCC. In addition, seven small-molecule candidates that may overcome the resistance of sorafenib were identified. Our findings provide a deeper and more comprehensive understanding of the occurrence and development of sorafenib resistance, along with some strategies and directions for improving the clinical efficacy of chemotherapeutic drugs.

## Data Availability

The original contributions presented in the study are included in the article/Supplementary Material, further inquiries can be directed to the corresponding author.

## Supplementary Material

Supplementary Figure S1 PPI Network.

Supplementary Table S1 Up-regulated and down-regulated genes after integration analysis.

Supplementary Table S2 GO enrichment analysis of the DEGs.

Supplementary Table S3 PPI Network Analysis.

Supplementary Table S4 List of hub genes.

Supplementary Table S5 Raw data for Figure 6.
