# Supplementary figures and images for: Integrated Transcriptomic Analysis Revealed Hub Genes and Pathways Involved in Sorafenib Resistance in Hepatocellular Carcinoma
Source: Pathol Oncol Res. 2021 Oct 19;27:1609985. doi: 10.3389/pore.2021.1609985 (PMC8560649; doi:10.3389/pore.2021.1609985)

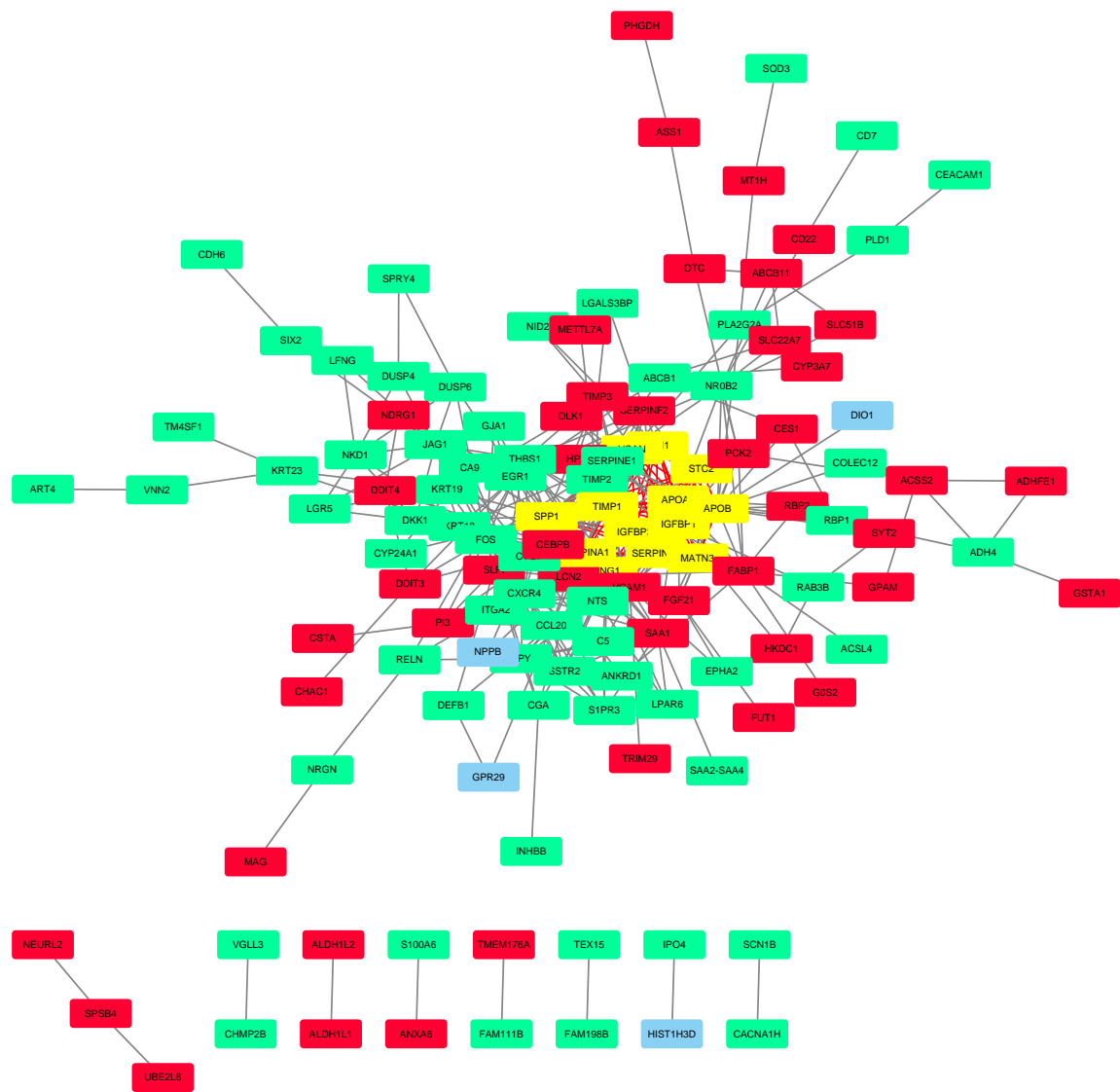

Supplement: Supplementary file 6 [file Image1.pdf]
